# Chemoprophylaxis effect of EGCG on various digestive system diseases: a systematic review and meta-analysis

**DOI:** 10.3389/fmed.2026.1809860

**Published:** 2026-06-10

**Authors:** Yanan Zhao, Zhongyu Wang, Mengchao Xu, Meilin Wang, Fanke Wang, Hongfei Pang, Yuhang Liu, Xintong Li, Haixiao Li, Wenya Zhang, Han Song, Ruizhe Shi, Jie Lin, Zhen Wang, Yuanyuan Wang, Haibo Jiang

**Affiliations:** 1 The First Hospital of Hebei Medical University, Shijiazhuang, China; 2 Xiongan Magic Medical Laboratory, Baoding, China; 3 School of Medicine, Hebei University of Engineering, Handan, Hebei, China; 4 School of Life Sciences and Food Engineering, Hebei University of Engineering, Handan, Hebei, China; 5 School of Basic Medicine, Hebei Medical University, Shijiazhuang, Hebei, China

**Keywords:** digestive system diseases, effects, epigallocatechin-3-gallate, mechanisms, progress, toxicology

## Abstract

**Background:**

Epigallocatechin-3-gallate (EGCG) constitutes the main component of tea polyphenols found in tea leaves and has been found to have a positive therapeutic effect on various digestive system diseases. However, no systematic review has been conducted on the research progress and mechanisms of EGCG in relation to digestive system diseases, an its toxicity.

**Methods:**

We conducted a comprehensive literature search for preclinical studies from the inception of each database to 28th September 2025, including Embase, PubMed, Web of Science, China National Knowledge Infrastructure and Veipu Information. These studies were manually screened based on predefined criteria. A comprehensive literature review and meta-analysis were then performed in accordance with the Preferred Reporting Items for Systematic Reviews and Meta-Analyses (PRISMA) guidelines.

**Results:**

A total of 74 animal studies were initially included. Following screening, 63 studies (involving 738 animals) met the inclusion criteria for the meta-analysis. EGCG’s animal experiments in digestive system diseases primarily focus on tongue squamous cancer, colorectal cancer, liver cancer, ulcerative colitis, gastric cancer, and functional gastrointestinal disorders. EGCG also has positive effects on pancreatic cancer, radiation enteritis, hepatitis B, oral cancer, esophageal cancer, radiation-induced esophagitis, hepatitis C, acute pancreatitis, fatty liver, and cancer prevention. The potential common pathways include VEGF, EGFR, Notch, Bax/Caspase, Nrf2/UGTIA10, JAK/STAT, NF-κB, IGF/IGF-IR, Caspase-1, HIF-1α/VEGF, TGFβ/p-ERK/p-Smad1/2 and M1/M2 cell polarization. EGCG suppresses cell proliferation through the induction of apoptosis; however, its underlying mechanisms warrant further investigation. Administration of high-dose EGCG alone can induce hepatotoxicity, an effect that is exacerbated under inflammatory conditions. In the context of diabetes, EGCG may also lead to nephrotoxicity. It should be noted that these toxic doses substantially exceed the levels typically attained through normal dietary consumption of tea. The mechanisms responsible for EGCG-mediated toxicity remain to be fully elucidated.

**Conclusion:**

*In vivo* studies have indicated the potential efficacy of EGCG in managing gastrointestinal diseases. However, further investigations are necessary to validate its therapeutic benefits, elucidate the underlying mechanisms, and assess its potential toxicity.

## 1 Introduction

Green tea, derived from the leaves of Camellia sinensis, is one of the most widely consumed beverages worldwide. Its health benefits have been attributed to polyphenolic compounds, particularly catechins. The major catechins in green tea include epicatechin (EC), epigallocatechin (EGC), epicatechin-3-gallate (ECG), and epigallocatechin-3-gallate (EGCG), among which EGCG is the most abundant and biologically active component (1, 2). EGCG is typically extracted from green tea using solvents such as water, ethanol, methanol, or their mixtures, often under controlled temperature conditions to preserve its stability (chemical information of EGCG is shown in Supplementary Figure 1). EGCG possesses a variety of pharmacological effects (3), including anti-inflammatory (4), antibacterial (5), antitumor (6, 7), lipid-lowering (8), and anti-Alzheimer’s disease (9), properties. In digestive system diseases specifically, EGCG has been demonstrated to have beneficial effects on conditions such as liver cancer (10, 11), colitis (12), gastric cancer, and colorectal cancer. However, EGCG also shows potential side effects, including hepatotoxicity (13), cardiotoxicity (14), and nephrotoxicity (15–17).

Previous reviews on EGCG have focused on its antitumor activity (18), derivatives (19), carriers (20) and bioavailability (21). Meta-analyses on EGCG in digestive system diseases have been limited to specific conditions, such as chemoprevention of colorectal cancer recurrence (22), inflammatory bowel disease (23), and non-alcoholic fatty liver disease (24). However, a comprehensive systematic review of EGCG’s research progress and mechanisms across different digestive system diseases—including whether common mechanisms exist—is lacking. Furthermore, whether EGCG has significant toxicity remains controversial, as evidenced by 11 animal studies reporting hepatotoxicity, cardiotoxicity, or nephrotoxicity (16, 25–34), while others have suggested no significant toxicity (25, 35).

These issues have not yet been systematically reviewed according to the PRISMA guidelines. Based on this, we conducted a systematic review of the progress and mechanisms of EGCG in digestive diseases and its side effects, with the aim of providing a reference for future research in this field.

## 2 Materials and methods

The included studies underwent qualitative and quantitative analysis in accordance with the Preferred Reporting Items for Systematic Review and Meta-analysis (PRISMA) guidelines (36).

### 2.1 Information sources and search strategy

For the literature search, a total of five databases were utilized: Embase, PubMed, Web of Science, China National Knowledge Infrastructure and Veipu Information. The literature search covers the period from the database’s inception to September 28, 2025. Literature search strategies are described in Supplementary Material 1.

### 2.2 Inclusion and exclusion criteria

The criteria for including and excluding literature were developed based on the ARRIVE guidelines^1^ for cell assays.

Inclusion criteria: (a) Experimental studies related to the effects of EGCG in digestive system diseases. (b) The study subjects were disease models related to digestive system diseases, and its side effects. (c) The experimental group only used the EGCG. (d) The control group served as the model group in the experiment. (e) The study findings encompassed the impact of EGCG on various signaling proteins and provided an overview of the associated mechanisms.

Exclusion criteria: (a) Studies that are not related to digestive system diseases. (b) The control group was not utilized. (c) Literature review, systematic review, thesis, and meta-analysis. (d) The research on EGCG derivatives. (e) No relevant results were reported. (f) The relevant or sufficient data could not be extracted. (g) Studies with incorrect conclusions or inadequate descriptions of outcomes. (h) Not *in vivo* studies.

### 2.3 Study selection and data extraction

We conducted the literature search using the literature search strategy. We independently reviewed studies that likely met the inclusion criteria, carefully documented their findings, and completed the flow diagram of the literature selection process. We extracted data based on the characteristics of the experimental type. The information extracted from animal experiments is as follows: first author/year, disease type/stimulation, animal/organ, intervention/dose/duration, outcomes of the *in vitro* study, outcomes of the *in vivo* study, and conclusions. All discrepancies were resolved through consultation with third parties.

### 2.4 Risk assessment of bias for included studies

SYstematic Review Centre for Laboratory animal Experimentation (SYRCLE’s) bias risk assessment tool (37) was utilized to assess the methodological quality of animal experiments. Two reviewers independently assessed the quality of the research, and any disagreements were resolved through communication with a third party.

The Animal Experimental Bias Assessment Tool evaluates study quality based on 10 criteria, including random sequence generation, baseline characteristic similarity, allocation concealment, random housing of animals, random selection of animals for outcome assessment, blinding of investigators, blinding of outcome assessors, incomplete outcome data, selective outcome reporting, and other potential sources of bias.

### 2.5 Statistical analysis and signal pathway detection

We performed a qualitative assessment of the animal studies included in this research. Furthermore, quantitative synthesis was conducted on animal experimental data deemed suitable for meta-analysis. Continuous outcomes were expressed as standardized mean differences (SMDs) with 95% confidence intervals. In cases where I^2^ exceeded 50% and the *P*-value was less than 0.05, a random-effects model was applied for meta-analysis. Data extraction from figures was carried out using Engauge Digitizer software, and meta-analyses of outcomes derived from animal experiments were performed using Review Manager version 5.3. Based on the experimental findings, we summarized the potential mechanisms of EGCG in the treatment of various digestive system diseases.

## 3 Results

### 3.1 Retrieval results of literature

**FIGURE 1:**
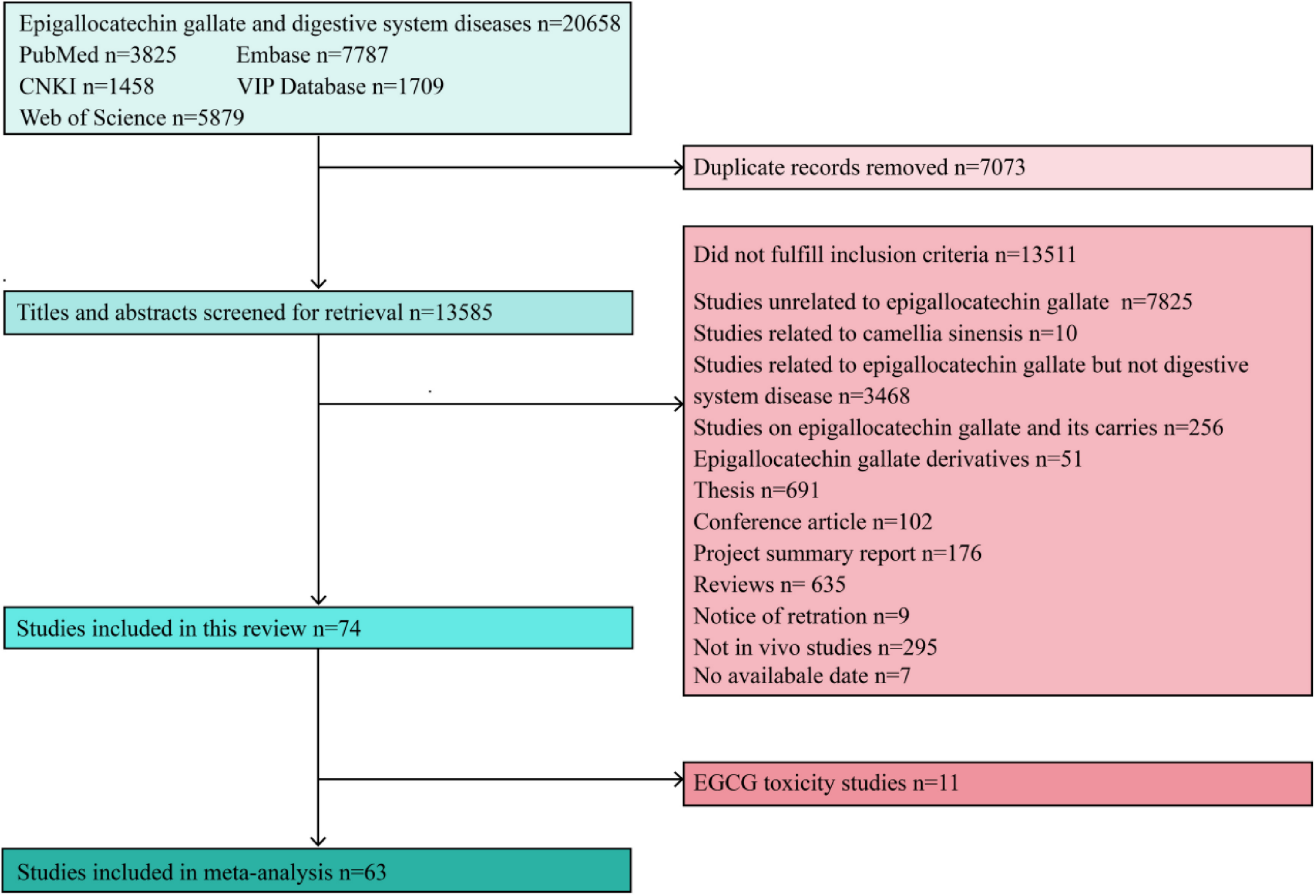
Flow diagram of the selection process.

We obtained 20,658 studies by searching five databases, and 7,073 studies remained after removing the 13,585 duplicates. After reviewing the titles and abstracts, a total of 13,223 studies were eliminated, resulting in 362 potential studies that could be considered for this research. After a full-text review, 74 studies were included in this systematic review. The flow diagram depicting the study selection process according to the preferred reporting standards is provided in Figure 1.

### 3.2 Description of the included studies

**FIGURE 2:**
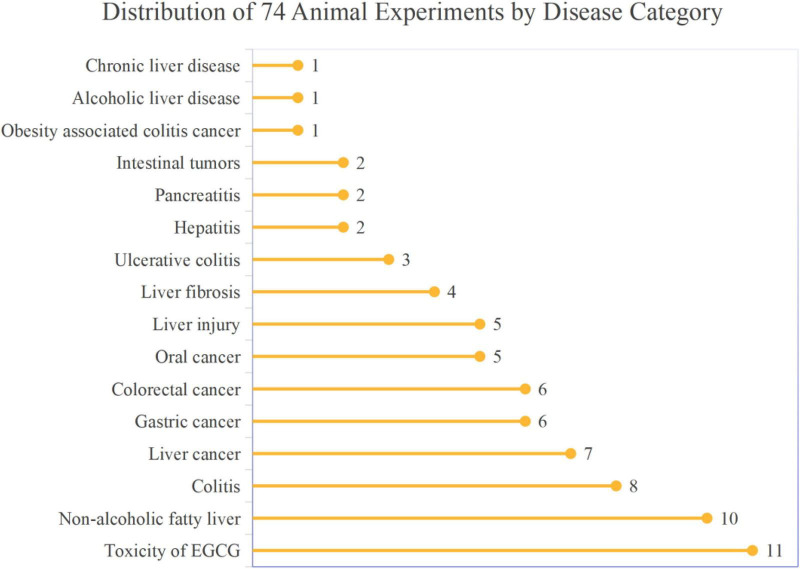
Distribution of 74 animal experiments by disease category.

A total of 74 animal experiments were included in this study (Figure 2). We distinguish different articles published by the same author in the same year by presenting them in the format of author plus year plus “a or b.” Of the 74 animal experiments included, there are five studies on oral cancer (38–42), six studies on gastric cancer (43–48), three studies on ulcerative colitis (49–51), eight studies on colitis (52–59), six studies on colorectal cancer (60–65), one study on obesity associated colitis cancer (66), 10 studies on non-alcoholic fatty liver (67–76), five studies on liver injury (77–81), seven studies on liver cancer (82–88), four studies on liver fibrosis (89–92), two studies on hepatitis (93, 94), one study on alcoholic liver disease (95), one study on chronic liver disease (96), two studies on pancreatitis (97, 98), two studies on the formation of intestinal tumors (99, 100), 11 studies on toxicity of EGCG (16, 25–34).

### 3.3 Risk of bias in the included studies

Out of the 63 animal experiment studies included, seven studies did not mention randomization in grouping. Therefore, the random sequence and allocation concealment of these studies are considered high risk. Among them, there were one study on oral cancer (38), one study on the formation of intestinal tumors (99), one study on obesity associated colitis cancer (66), two studies on non-alcoholic fatty liver (70, 72), one study on liver injury (78), one study on chronic liver disease (95).

There were 51 studies that conducted randomized grouping. However, the method of randomization was not described in detail, so the risks of randomization and allocation concealment for these 51 studies are unclear. Among them, there were three studies on oral cancer (39, 41, 42), five studies on gastric cancer (43–46, 48), one study on the formation of intestinal tumors (100), six studies on colorectal cancer (60–65), eight studies on colitis (52–59), three studies on ulcerative colitis (49–51), eight studies on non-alcoholic fatty liver (67–69, 71, 72), three studies on liver injury (77, 79, 80), seven studies on liver cancer (82–88), four studies on liver fibrosis (89–92), 2 studies on hepatitis (93, 94), one study on alcoholic liver disease (95).

**FIGURE 3:**
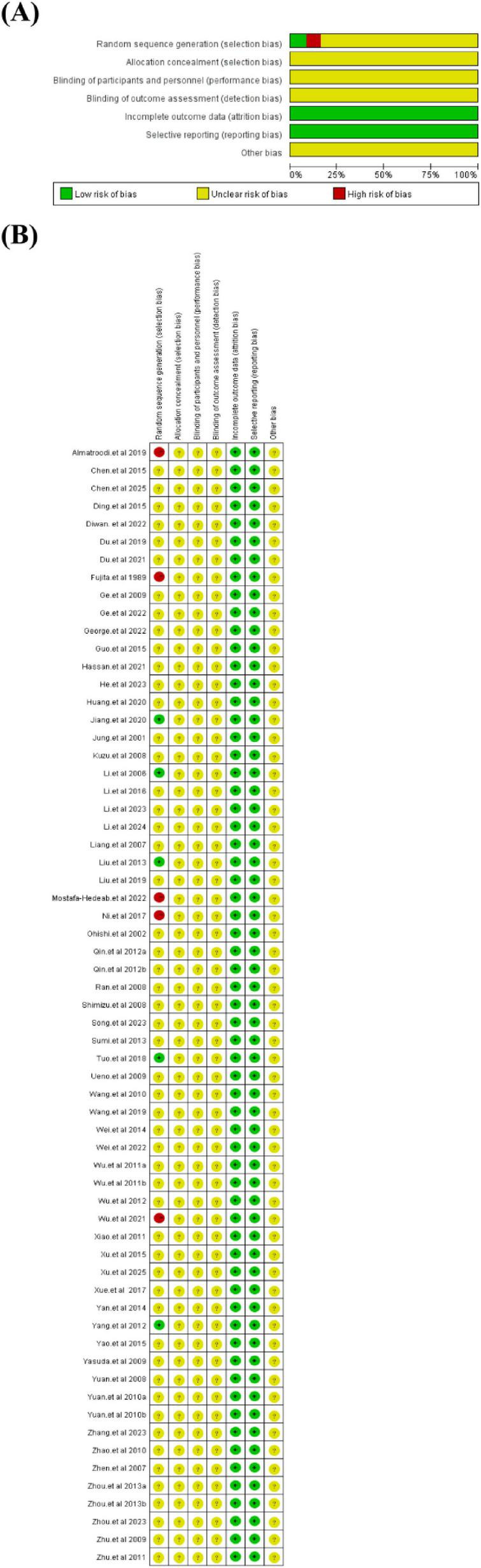
Risk of bias assessments **(A)** and summary **(B)** of the included studies.

Five studies used the random number table method for grouping (40, 47, 81, 97, 98). All 63 studies included in the reports were complete, and there was no selective reporting. Nevertheless, the uncertainty regarding bias in the analysis of the 63 studies remains regarding randomization of animals, blinding of researchers, and other possible sources of bias (Figure 3).

### 3.4 Effects of tumor size

#### 3.4.1 Oral cancer

**FIGURE 4:**
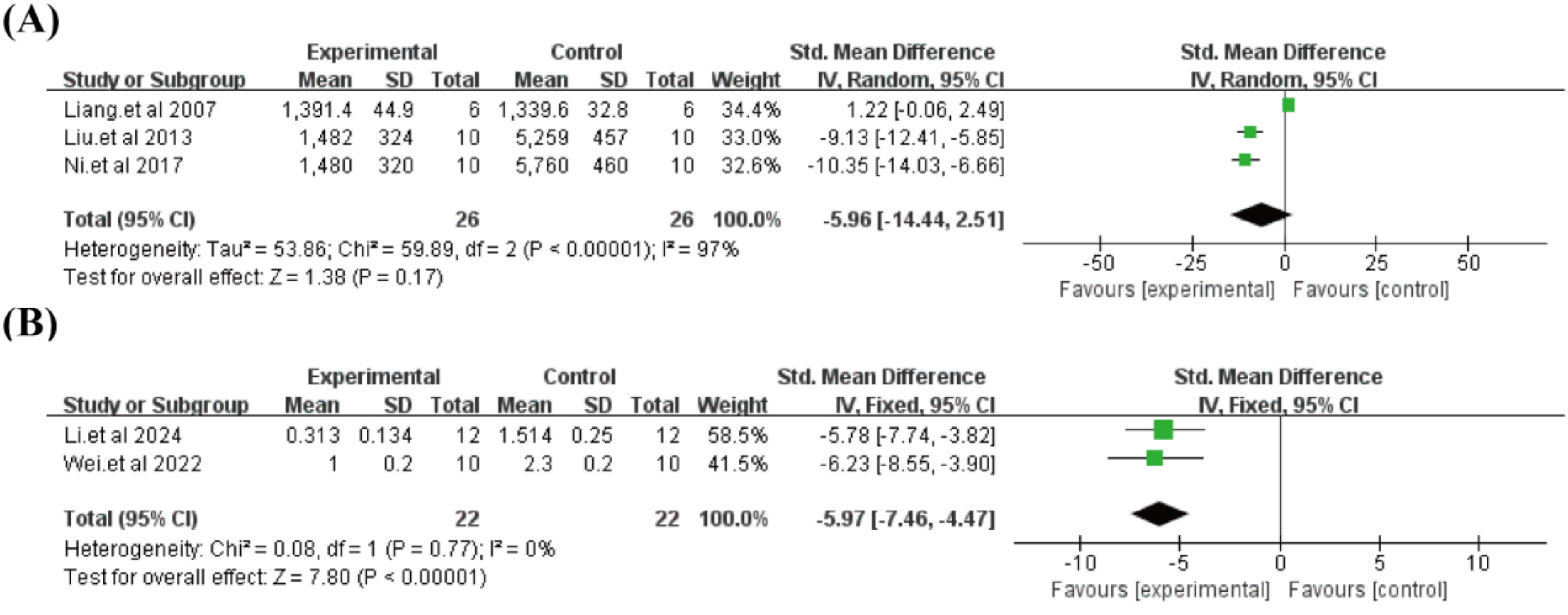
Forest plot showing the association between epigallocatechin-3-gallate (EGCG) and tumor size in KB xenograft models. **(A)** Tumor weight in KB xenograft models (fixed-effects model): SMD = –5.96 (95% CI: –14.44, 2.51); **(B)** Tumor volume in KB xenograft models (fixed-effects model): SMD = –5.97 (95% CI: –7.46, –4.47). Effect sizes (SMD) and 95% CIs for each study are shown as squares (size proportional to study weight) and horizontal lines. The pooled effect size is represented by a diamond. KB refers to a human oral epidermoid carcinoma cell line.

A total of five studies on oral cancer were included. Three studies investigated the effect of EGCG on tumor volume and weight of the KB xenograft model (38–40). According to the meta-analysis using the random effects model, EGCG reduced the weight [*n* = 52, SMD = −5.96, 95% CI (−14.44, 2.51), *p* = 0.17, I^2^ = 97%] (Figure 4A) and volume [*n* = 52, SMD = −6.13, 95% CI (−13.65, 1.39), *p* = 0.11, I^2^ = 96%] (Figure 4B) of KB xenograft tumors. Another two studies investigated the effect of EGCG on tumor weight (41, 42). According to the meta-analysis using the fixed effects model, EGCG significantly reduced the weight of oral cancer [*n* = 44, SMD = −5.97, 95% CI (−7.46, −4.47), *p* < 0.00001, I^2^ = 0%] (Supplementary Figure 2). EGCG reduced the tumor volume of the KB xenograft model that was resistant to vincristine (VCR) and reduced the expression of low-density lipoprotein receptor-related protein (LRP) (39).

#### 3.4.2 Gastric cancer

A total of six studies on gastric cancer were included. Four studies investigated the effect of EGCG on tumor volume of the BGC-823 or SGC7901 xenograft model (43–45, 47). EGCG significantly reduced the volume of gastric cancer [BGC-823: *n* = 40, SMD = −3.84, 95% CI (−4.97, −2.71), *p* < 0.00001, I^2^ = 0%; SGC7901: *n* = 22, SMD = −22.03, 95% CI (−37.52, −6.54), p = 0.005, I^2^ = 68%] (Supplementary Figures 3A,B). Three studies investigated the effect of EGCG on tumor weight of the SGC7901 xenograft model (44, 46, 47). EGCG reduced the weight of gastric cancer [*n* = 36, SMD = −6.39, 95% CI (−10.63, −2.16), *p* = 0.003, I^2^ = 78%] (Supplementary Figure 3C).

#### 3.4.3 Colorectal cancer

A total of four studies assessed the tumor size of the HT29 xenograft model levels in colorectal cancer animals (60–63). EGCG reduced the volume [*n* = 40, SMD = −3.24, 95% CI (−5.79, −0.69), *p* = 0.01, I^2^ = 89%] (Supplementary Figure 4A) and weight [*n* = 60, SMD = −2.19, 95% CI (−4.68, 0.30), *p* = 0.08, I^2^ = 87%] (Supplementary Figure 4B) of colorectal cancer.

#### 3.4.4 Liver cancer

A total of six studies on gastric cancer were included. Four studies assessed the tumor size of the HepG2 xenograft model and two studies of SMMC-7721 (82–86, 88). EGCG significantly reduced the weight of liver cancer [HepG2: *n* = 54, SMD = −3.68, 95% CI (−4.69, −2.67), *p* < 0.00001, I^2^ = 41%; SMMC-7721: *n* = 32, SMD = −3.47, 95% CI (−7.22, 0.28), *p* = 0.07, I^2^ = 85%] (Supplementary Figures 5A,B). Moreover, EGCG inhibited the increase in tumor volume of HepG2 xenograft model [*n* = 42, SMD = −3.53, 95% CI (−6.72, −0.35), *p* = 0.01, I^2^ = 88%] (Supplementary Figure 5C).

### 3.5 Effects of microvessel density

#### 3.5.1 Gastric cancer

Five studies assessed the impact of EGCG on the levels of microvessel density (MVD) in BGC-823 or SGC7901 xenograft model (43–46, 48). The EGCG treatment reduced MVD levels [BGC-823: *n* = 60, SMD = −7.36, 95% CI (−9.67, −5.04), *p* < 0.00001, I^2^ = 53%; SGC7901: *n* = 26, SMD = −4.79, 95% CI (−11.67, 2.10), *p* = 0.07, I^2^ = 89%] (Supplementary Figures 6A,B).

#### 3.5.2 Liver cancer

Two studies assessed the impact of EGCG on levels of MVD in HepG2 xenograft model (83, 87). EGCG significantly reduced MVD levels [*n* = 28, SMD = −7.29, 95% CI (−11.38, −3.21), *p* = 0.0005, I^2^ = 62%] (Supplementary Figure 6C).

### 3.6 Effects of liver function

#### 3.6.1 AST and ALT

**FIGURE 5:**
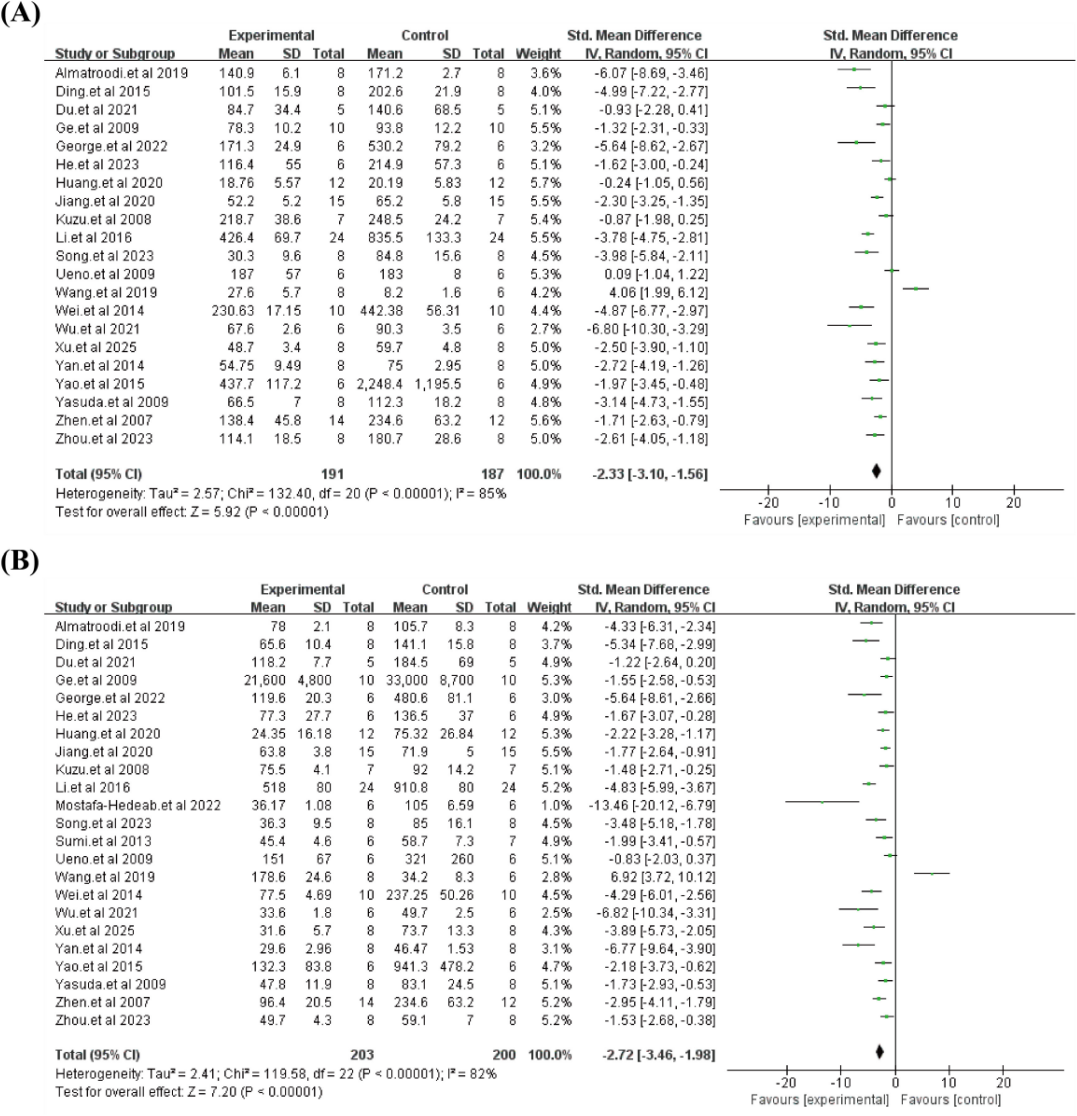
Forest plot showing the association between epigallocatechin-3-gallate (EGCG) and liver enzyme levels in xenograft models. **(A)** AST (aspartate aminotransferase; random-effects model): SMD = –2.33 (95% CI: –3.10, –1.56); **(B)** ALT (alanine aminotransferase; random-effects model): SMD = –2.72 (95% CI: –3.46, –1.98). Effect sizes (SMD) and 95% CIs for each study are shown as squares and horizontal lines; the diamond represents the pooled effect size. Heterogeneity is indicated by I^2^ statistics shown in each panel.

Twenty-one studies assessed the impact of EGCG on the levels of AST in animals (68, 70, 71, 74–77, 80, 81, 89, 91–94, 101). The EGCG treatment significantly reduced AST levels [*n* = 378, SMD = −2.33, 95% CI (−3.10, −1.56), *p* < 0.00001, I^2^ = 85%] (Figure 5A). Furthermore, twenty-three studies assessed the impact of EGCG on the levels of ALT in animals (67–81, 89–96), revealing that EGCG treatment significantly reduced ALT levels [*n* = 403, SMD = −2.72, 95% CI (−3.46, −1.98), *p* < 0.00001, I^2^ = 82%] (Figure 5B).

### 3.7 Effects of other indicators

#### 3.7.1 Disease activity index score

**FIGURE 6:**
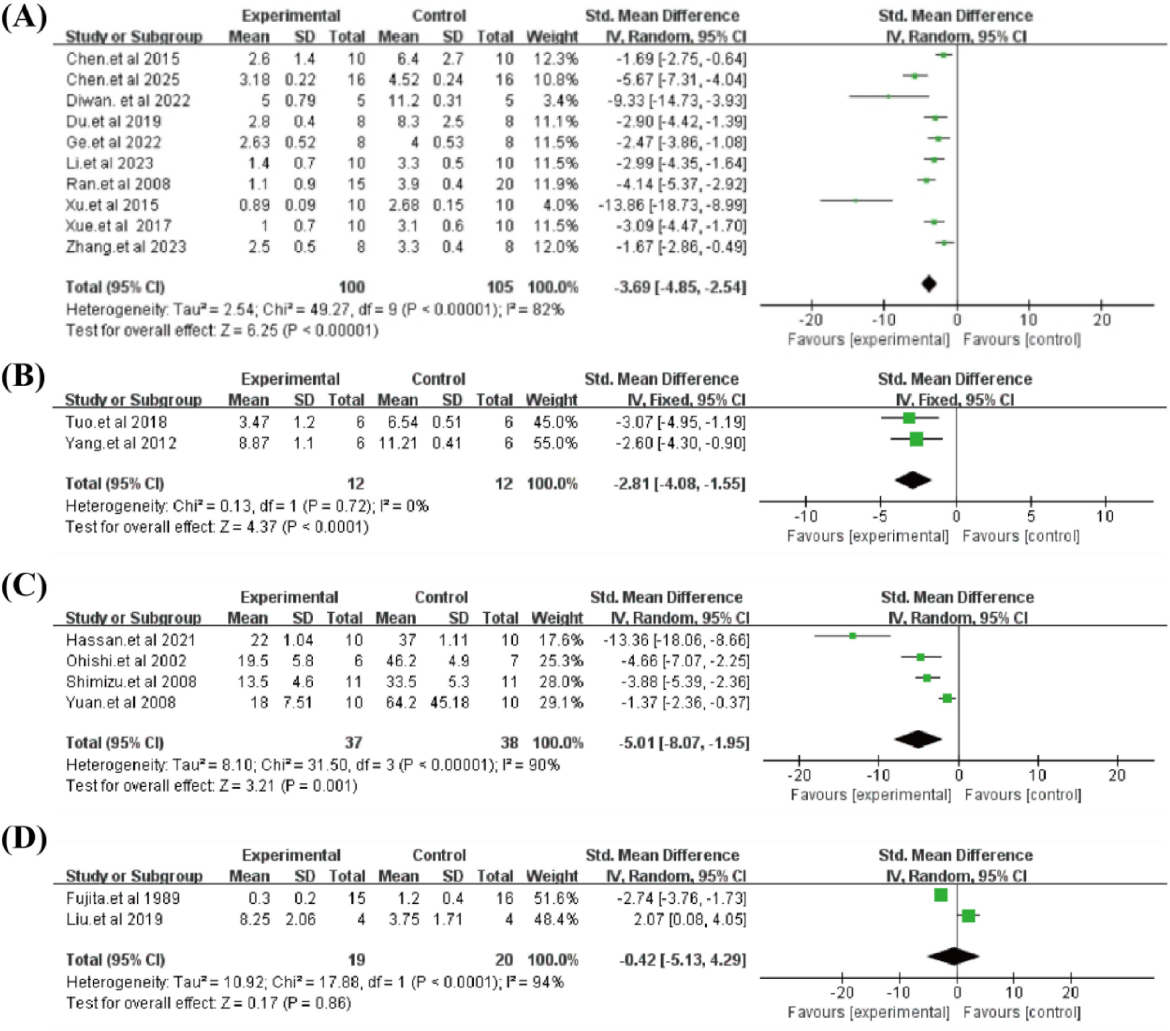
Forest plot showing the association between epigallocatechin-3-gallate (EGCG) and key efficacy endpoints. **(A)** Disease activity index (DAI) score (random-effects model): SMD = –3.69 (95% CI: –4.85, –2.54); **(B)** Pathological score (random-effects model): SMD = –2.81 (95% CI: –4.08, –1.55); **(C)** aberrant crypt foci (ACF) (random-effects model): SMD = –5.01 (95% CI: –8.07, –1.95); **(D)** Tumor number (random-effects model): SMD = –0.42 (95% CI: –5.13, 4.29). Effect sizes (SMD) and 95% CIs for each study are shown as squares and horizontal lines; the diamond represents the pooled effect size. Heterogeneity is indicated by I^2^ statistics shown in each panel.

Ten experimental groups investigated the effect of EGCG on the disease activity index (DAI) score (49–58). EGCG had a significant impact on reducing DAI scores [*n* = 205, SMD = −3.69, 95% CI (−4.85, −2.54), *p* < 0.00001, I^2^ = 82%] (Figure 6A).

#### 3.7.2 Pathological score

Two studies assessed the impact of EGCG on the pathological score (99, 100). The EGCG significantly reduced pathological score [*n* = 24, SMD = −2.81, 95% CI (−4.08, −1.55), *p* < 0.00001, I^2^ = 0%] (Figure 6B).

#### 3.7.3 Aberrant crypt foci

Four experimental groups investigated the effect of EGCG on the aberrant crypt foci (ACF). EGCG had a significant impact on reducing ACF [*n* = 75, SMD = −5.01, 95% CI (−8.07, −1.95), *p* < 0.00001, I^2^ = 90%] (Figure 6C).

#### 3.7.4 Number of tumors

Two experimental groups investigated the effect of EGCG on the number of tumors. EGCG didn’t reduce the number of tumors [*n* = 39, SMD = −0.42, 95% CI (−5.13, 4.29), *p* = 0.86, I^2^ = 94%] (Figure 6D).

## 4 Progress of the toxicity of EGCG

### 4.1 Hepatotoxicity

Seven studies indicated using high doses of EGCG can cause liver toxicity (26, 28–33). The mechanism of EGCG-triggered hepatotoxicity involves suppression of major antioxidant enzymes, and the nuclear factor erythroid 2-related factor 2 (Nrf2) rescue pathway plays a vital role for counteracting EGCG toxicity (30–32). It was observed that the dose of EGCG that caused toxicity was far higher than that normally consumed through drinking tea. In the presence of inflammation, high doses of EGCG exhibit more pronounced hepatotoxicity (29). One animal experiment found that supplementing with copper ions can reduce the hepatotoxicity of EGCG (33).

### 4.2 Other outcomes

One study found that EGCG with no genotoxic *in vitro* (27). One study indicated that EGCG may cause nephrotoxicity and cardiotoxicity in diabetic mice (16). Another study had indicated that excessive intake of EGCG may lead to an increase in blood lipids, and the specific mechanism requires further experimental research (28). Two studies indicated that EGCG has no significant toxicity, but applying EGCG while using topical hair removal cream can cause skin toxicity (25, 34)

## 5 Discussion

### 5.1 Progress and mechanisms of EGCG in digestive system diseases in animal experiments

Studies on diseases of the digestive system *in vivo* focus on various conditions. These include oral cancer (five studies), gastric cancer (six studies), ulcerative colitis (three studies), colitis (eight studies), colorectal cancer (six studies), obesity associated colitis cancer (one study), non-alcoholic fatty liver (10 studies), liver injury (five studies), liver cancer (seven studies), liver fibrosis (four studies), hepatitis (two studies), alcoholic liver disease (one study), chronic liver disease (one study), pancreatitis (two studies), the formation of intestinal tumors (two studies). The underlying mechanisms involved in these *in vivo* studies are presented in Table 1.

**TABLE 1:** Mechanisms and pathways involved in the animal experiments.

Disease type	Cell lines	Mechanisms and pathways involved in the animal experiments
Oral cancer	KB	NA
Gastric cancer	BGC-823	VEGF
	SGC7901	Bcl-2/Bax, Caspase-3, VEGF, STAT3
Colorectal cancer	HT29	Nrf2/UGT1A, VEGF, p53
Liver cancer	HepG2	HIF-1α/VEGF, bFGF
	SMMC7721	HIF-1α/VEGF
Obesity associated colitis cancer	NA	IGF/IGF-IR
Ulcerative colitis	NA	TLR4/MyD88/NF-B, IL-6, Treg/Th17, NLPR3
Colitis	NA	JAK2/STAT3, NF-κB, regulate gut microbiota, inflammatory bowel disease, TNFα/IL-6/IL-1β, Caspase-1, Nrf2/GPX4
Liver fibrosis	NA	MT1-MMP/MMP-2, PDGFRβ/IGF-1R
Liver injury	NA	PI3K/AKT
Alcoholic liver disease	NA	ALD
Chronic liver disease	NA	TGFβ/p-ERK/p-Smad1/2
Pancreatitis	NA	NF-κB, TNF-α

Bcl-2, B cell lymphoma 2; Bax, BCL2-associated X protein; STAT3, signal transducer and activator of transcription 3; HIF-1α, hypoxia-inducible factor; IGF, insulin like growth factor; TLR4, toll-like receptor 4; NF-B, nuclear factor kappa-B; IL-6, interleukin- 6; NLPR3, NOD-like receptor thermal protein domain associated protein 3; JAK2, janus kinase 2; NF-κB, non-canonical nuclear factor-kappaB; MT1, melatoninreceptor 1A; MMP, matrix metalloproteinases; PDGFRβ, platelet-derived growth factor receptor beta; PI3K, phosphoinositide 3-kinase; AKT, protein kinase B; ALD, atomic layer deposition; TNF-α, tumor necrosis factor-alpha.

In cancer models (oral, gastric, colorectal, and liver cancer), the observed reductions in tumor size, tumor weight, and MVD can be explained by EGCG’s well-established multi-target anticancer mechanisms. EGCG inhibits tumor cell proliferation by downregulating the PI3K/Akt and ERK signaling pathways, induces apoptosis through caspase-3 activation and Bcl-2 family modulation, and suppresses angiogenesis by reducing VEGF expression. The significant MVD reduction observed in gastric and liver cancer models (SMD = −7.36 to −4.79) specifically supports EGCG’s anti-angiogenic activity.

In non-cancer conditions such as colitis, non-alcoholic fatty liver disease, and liver injury, EGCG exerts protective effects primarily through anti-inflammatory and antioxidant pathways. EGCG suppresses NF-κB activation, reducing pro-inflammatory cytokines (TNF-α, IL-6, IL-1β). Its antioxidant activity is mediated by activation of the Nrf2/HO-1 pathway, scavenging reactive oxygen species, and restoring endogenous antioxidant enzymes (SOD, GPx). The significant reductions in AST and ALT levels (SMD = −2.33 and −2.72) reflect EGCG’s hepatoprotective effects against chemical- or diet-induced liver injury. In colitis models, the decreased DAI and pathological scores (SMD = −3.69 and −2.81) are attributable to EGCG’s preservation of intestinal barrier integrity via upregulation of tight junction proteins (occludin, claudin, ZO-1) and modulation of gut microbiota.

Despite evidence that EGCG inhibits gastrointestinal tumor cell proliferation, invasion, and migration *in vivo*, its effect on tumorigenesis remains controversial. In the present meta-analysis, tumor size and tumor number were selected as measures of anti-proliferative effects; MVD as an indicator of anti-angiogenic activity (via the VEGF pathway) (48); and ACF as a surrogate biomarker of colon carcinogenesis (59). Regarding intestinal carcinogenesis, the included studies yielded conflicting results: Fujita et al. reported that EGCG inhibits tumor promotion (102), Liu et al. found that EGCG promotes colorectal adenoma formation (100), while Ohishi et al. observed inhibition of colon carcinogenesis (65). Thus, the preventive effect of EGCG on intestinal carcinogenesis remains inconclusive and requires further investigation.

In experimental models of colitis, EGCG has been demonstrated to strengthen tight junctions, promote cellular autophagy, and exert anti-inflammatory as well as antioxidant activities. To quantify these effects, we selected the DAI and pathological score, which are validated composite measures of inflammation severity and tissue damage in colitis models. Additionally, EGCG modulates the gut microbiota and helps preserve intestinal barrier integrity, thereby ameliorating the pathology of colitis.

For liver-related studies, AST and ALT were selected as sensitive indicators of hepatocyte injury, reflecting both EGCG’s hepatoprotective effects and its potential hepatotoxicity. The imbalance of gut microbiota is associated with various digestive system diseases, such as pancreatic cancer, liver cancer, gastric cancer, functional gastrointestinal disorders, and gastritis (101–105). EGCG may have a beneficial effect on liver injury and colitis by regulating the gut microbiota (52, 79). However, it is still unclear whether EGCG can have a beneficial effect on other digestive system diseases by regulating the intestinal flora. This could be a potential area for further research.

Due to the limited number of studies included on pancreatitis, gastric cancer, oral cancer, and hepatitis, further research is needed to demonstrate the impact of EGCG on these disease models.

### 5.2 Toxicity of EGCG

We included 11 studies on EGCG toxicity. Whether EGCG exhibits cardiotoxicity, hepatotoxicity, and nephrotoxicity is still a matter of debate. The experimental protocols of the one studies on cardiotoxicity may also be inappropriate (16). Therefore, more experiments with a well-designed experimental protocol are needed to study the toxicity of EGCG. Before the toxicity of EGCG has been defined, caution is required for its clinical use.

### 5.3 The value and limitations of this study

This systematic review summarizes the therapeutic progress of EGCG in digestive system diseases and elucidates its potential underlying mechanisms. The findings provide a valuable reference for future research. However, this study has several limitations. Some of the included animal experiments exhibited certain biases, and five studies were assessed as having a high risk of bias in random sequence generation, which may compromise the quality and reliability of the conclusions. Another limitation is the notable scarcity of studies investigating the role of EGCG in several specific conditions, including oral, esophageal, pancreatic, and nasopharyngeal cancers, and liver fibrosis, which hinders definitive conclusions.

## 6 Conclusion

This meta-analysis of 74 animal experiments quantifies EGCG’s therapeutic effects: tumor size reduction in oral, gastric, colorectal, and liver cancers (SMD: −6.13 to −2.19); MVD reduction in gastric and liver cancers (SMD: −7.36 to −4.79); improved liver function (AST: SMD = −2.33; ALT: SMD = −2.72); reduced DAI (SMD = −3.69) and pathological scores (SMD = −2.81) in colitis; and reduced ACF (SMD = −5.01) for colon cancer chemoprevention. However, EGCG did not significantly reduce tumor number (SMD = −0.42, *p* = 0.86), and its effect on intestinal carcinogenesis remains inconclusive.

High-dose EGCG causes hepatotoxicity via Nrf2 pathway suppression, with inflammation as an exacerbating factor. No genotoxicity was found.

This study provides the first quantitative effect size benchmarks for EGCG in digestive disease models, identifies specific research gaps (intestinal tumor number, hepatotoxicity threshold, understudied diseases), and offers evidence-based guidance for dose selection in future preclinical research.

## Data Availability

Publicly available datasets were analyzed in this study. This data can be found here: the datasets analyzed during the current study are available from the corresponding primary studies included in this systematic review, as detailed in the reference list. No specific repository or accession numbers are applicable, as the data were extracted from published articles.

## Footnotes

1
https://arriveguidelines.org/
