# Supplementary material for: Chemoprophylaxis effect of EGCG on various digestive system diseases: a systematic review and meta-analysis
Source: Front Med (Lausanne). 2026 Jun 10;13:1809860. doi: 10.3389/fmed.2026.1809860 (PMC13290675; doi:10.3389/fmed.2026.1809860)
Supplement: Supplementary file 1 [file Supplementary_file_1.docx]

**Annex - Supplementary Items**

**Supplementary Material 1: Search strategies in PubMed，Web of science and Embase**

**PubMed**

# 1 EGCG

# 2 Epigallocatechin-3-gallate

# 3 (# 1 OR # 2)

(EGCG) OR (Epigallocatechin-3-gallate)

# 4 Mouth

# 5 Oral Cavity

# 6 Mouth Diseases

# 7 Oral disease

# 8 Tongue

# 9 Tongue disease

# 10 (#4 OR #5 OR #6 OR #7 OR # 8 OR #9 )

# 11 (# 3) AND (# 10)

((EGCG) OR (Epigallocatechin-3-gallate)) AND (((((((Mouth)) OR (Oral Cavity)) OR (Mouth Diseases)) OR (Oral disease)) OR (Tongue)) OR (Tongue disease))

# 12 Esophagus

# 13 Esophageal Neoplasms

# 14 Esophagus Cancer

# 15 Esophageal Diseases

# 16 Esophagitis

# 17 (#12 OR # 13 OR # 14 OR # 15 OR # 16)

# 18 (# 3) AND (# 17)

((EGCG) OR (Epigallocatechin-3-gallate)) AND (((((Esophagus) OR (Esophageal Neoplasms)) OR (Esophagus Cancer)) OR (Esophageal Diseases)) OR (Esophagitis))

# 19 Stomach

# 20 Gastric Cancer

# 21 Stomach Neoplasms

# 22 Stomach Ulcer

# 23 Gastric Ulcer

# 24 Stomach Diseases

# 25 Gastric Diseases

# 26 (#19 OR #20 OR #21 OR #22 OR # 23 OR #24 OR #25)

# 27 (# 3) AND (# 26)

((EGCG) OR (Epigallocatechin-3-gallate)) AND (((((((Stomach) OR (Gastric Cancer)) OR (stomach neoplasms)) OR (Stomach Ulcer)) OR (Gastric Ulcer)) OR (Stomach Diseases)) OR (Gastric Diseases))

# 28 Duodenum

# 29 Duodenal Neoplasms

# 30 Duodenal Diseases

# 31 Duodenal ulcer

# 32 (#28 OR #29 OR #30 OR #31)

# 33 (# 3) AND (# 32)

((EGCG) OR (Epigallocatechin-3-gallate)) AND ((((Duodenum) OR (Duodenal Neoplasms)) OR (Duodenal Diseases)) OR (Duodenal ulcer))

# 34 Jejunum

# 35 Jejunal Neoplasms

# 36 Jejunal Diseases

# 37 (#34 OR #35 OR #36)

# 38 (# 3 OR # 37 )

((EGCG) OR (Epigallocatechin-3-gallate)) AND (((Jejunum) OR (Jejunal Neoplasms)) OR (Jejunal Diseases))

# 39 Ileum

# 40 Ileal Neoplasms

# 41 Ileal Diseases

# 42 (#39 OR #40 OR #41)

# 43 (# 3 ) AND (# 42)

((EGCG) OR (Epigallocatechin-3-gallate)) AND (((Ileum) OR (Ileal Neoplasms)) OR (Ileal Diseases))

# 44 Colon

# 45 Colonic Neoplasms

# 46 Sigmoid Diseases

# 47 Colonic Diseases

# 48 Colonic cancer

# 49 Colitis

# 50 Colitis-Associated Neoplasms

# 51 Crohn Disease

# 52 Colitis, Ulcerative

# 53 Irritable Bowel Syndrome

# 54 (#44 OR #45 OR #46 OR #47 OR # 48 OR #49 OR #50 OR # 51 OR # 52 OR # 53 ) # 55 (# 3) AND (# 54)

((EGCG) OR (Epigallocatechin-3-gallate)) AND ((((((((((Colon) OR (Colonic Neoplasms)) OR (Sigmoid Diseases)) OR (Colonic Diseases)) OR (Colonic cancer)) OR (Colitis)) OR (Colitis-Associated Neoplasms)) OR (Crohn Disease)) OR (Colitis, Ulcerative)) OR (Irritable Bowel Syndrome))

# 56 Rectum

# 57 Rectal Neoplasms

# 58 Rectal disease

# 59 (#56 OR #57 OR #58)

# 60 (# 3) AND (# 59)

((EGCG) OR (Epigallocatechin-3-gallate)) AND (((Rectum) OR (Rectal Neoplasms)) OR (Rectal disease))

# 61(# 11 OR # 18 OR # 27 OR # 33 OR # 38 OR # 43 OR # 55 OR # 60)

# 62 Liver

# 63 Liver disease

# 64 Non-alcoholic Fatty Liver Disease

# 65 Liver Neoplasms

# 66 Hepatic Cancer

# 67 Hepatocellular Cancer

# 68 Fatty Liver

# 69 Hepatitis

# 70 Hepatic Fibrosis

# 71 (#62 OR #63 OR #64 OR #65 OR #66 OR #67 OR # 68 OR # 69 OR # 70)

# 72 (#3 AND # 71)

((EGCG) OR (Epigallocatechin-3-gallate)) AND (((((((((Liver) OR (Liver disease)) OR (Non-alcoholic Fatty Liver Disease)) OR (Liver Neoplasms)) OR (Hepatic Cancer)) OR (Hepatocellular Cancer)) OR (Fatty Liver)) OR (Hepatitis)) OR (Hepatic Fibrosis))

# 73 Pancreas

# 74 Pancreatic Neoplasms

# 75 Pancreas Cancer

# 76 Pancreatic Cancer

# 77 Pancreatitis

# 78 Pancreatic Diseases

# 79 (#73 OR #74 OR #75 OR #76 OR #77 OR #78)

# 80 (#3 AND # 79)

((EGCG) OR (Epigallocatechin-3-gallate)) AND ((((((Pancreas) OR (Pancreatic Neoplasms)) OR (Pancreas Cancer)) OR (Pancreatic Cancer)) OR (Pancreatitis)) OR (Pancreatic Diseases))

# 81 (#61 OR #72 OR # 80)

# 82 Toxicology

# 83 Toxicity

# 84 Side effects

# 85 (# 82 OR # 83 OR # 84)

# 86 ( # 85 AND # 3)

(((Toxicology) OR (Toxicity)) OR (Side effects)) AND ((EGCG) OR (Epigallocatechin-3-gallate))

# 87 Salivary Glands

# 88 Gland, Salivary

# 89 Submandibular Gland

# 90 Gland, Submandibular

# 91 Parotid Gland

# 92 Gland, Parotid

# 93 Sublingual Gland

# 94 Gland, Sublingual

# 95 (# 87 OR # 88 OR # 89 OR # 90 OR # 91 OR # 92 OR # 93 OR # 94)

# 96 (# 95 AND # 3)

((((((((Salivary Glands) OR (Gland, Salivary)) OR (Submandibular Gland)) OR (Gland, Submandibular)) OR (Parotid Gland)) OR (Gland, Parotid)) OR (Sublingual Gland)) OR (Gland, Sublingual)) AND ((EGCG) OR (Epigallocatechin-3-gallate))

# 97 Gastrointestinal Tract

# 98 Digestive Tract

# 99 Digestive System

# 100 Digestive glands

# 101 (# 97 OR # 98 OR # 99 OR # 100 )

# 102 (# 101 AND # 3)

((((Gastrointestinal Tract) OR (Digestive Tract)) OR (Digestive System)) OR (digestive glands)) AND ((EGCG) OR (Epigallocatechin-3-gallate))

# 103 (# 81 OR # 86 OR # 96 OR # 102)

((((((((((((((EGCG) OR (Epigallocatechin-3-gallate)) AND (((((((Mouth)) OR (Oral Cavity)) OR (Mouth Diseases)) OR (Oral disease)) OR (Tongue)) OR (Tongue disease))) OR (((EGCG) OR (Epigallocatechin-3-gallate)) AND (((((Esophagus) OR (Esophageal Neoplasms)) OR (Esophagus Cancer)) OR (Esophageal Diseases)) OR (Esophagitis)))) OR (((EGCG) OR (Epigallocatechin-3-gallate)) AND (((((((Stomach) OR (Gastric Cancer)) OR (stomach neoplasms)) OR (Stomach Ulcer)) OR (Gastric Ulcer)) OR (Stomach Diseases)) OR (Gastric Diseases)))) OR (((EGCG) OR (Epigallocatechin-3-gallate)) AND ((((Duodenum) OR (Duodenal Neoplasms)) OR (Duodenal Diseases)) OR (Duodenal ulcer)))) OR (((EGCG) OR (Epigallocatechin-3-gallate)) AND (((Jejunum) OR (Jejunal Neoplasms)) OR (Jejunal Diseases)))) OR (((EGCG) OR (Epigallocatechin-3-gallate)) AND (((Ileum) OR (Ileal Neoplasms)) OR (Ileal Diseases)))) OR (((EGCG) OR (Epigallocatechin-3-gallate)) AND ((((((((((Colon) OR (Colonic Neoplasms)) OR (Sigmoid Diseases)) OR (Colonic Diseases)) OR (Colonic cancer)) OR (Colitis)) OR (Colitis-Associated Neoplasms)) OR (Crohn Disease)) OR (Colitis, Ulcerative)) OR (Irritable Bowel Syndrome)))) OR (((EGCG) OR (Epigallocatechin-3-gallate)) AND (((Rectum) OR (Rectal Neoplasms)) OR (Rectal disease)))) OR (((EGCG) OR (Epigallocatechin-3-gallate)) AND (((((((((Liver) OR (Liver disease)) OR (Non-alcoholic Fatty Liver Disease)) OR (Liver Neoplasms)) OR (Hepatic Cancer)) OR (Hepatocellular Cancer)) OR (Fatty Liver)) OR (Hepatitis)) OR (Hepatic Fibrosis)))) OR (((EGCG) OR (Epigallocatechin-3-gallate)) AND ((((((Pancreas) OR (Pancreatic Neoplasms)) OR (Pancreas Cancer)) OR (Pancreatic Cancer)) OR (Pancreatitis)) OR (Pancreatic Diseases)))) OR ((((Toxicology) OR (Toxicity)) OR (Side effects)) AND ((EGCG) OR (Epigallocatechin-3-gallate)))) OR (((((((((Salivary Glands) OR (Gland, Salivary)) OR (Submandibular Gland)) OR (Gland, Submandibular)) OR (Parotid Gland)) OR (Gland, Parotid)) OR (Sublingual Gland)) OR (Gland, Sublingual)) AND ((EGCG) OR (Epigallocatechin-3-gallate)))) OR (((((Gastrointestinal Tract) OR (Digestive Tract)) OR (Digestive System)) OR (digestive glands)) AND ((EGCG) OR (Epigallocatechin-3-gallate)))

**Embase**

((((((((((((((EGCG) OR (Epigallocatechin-3-gallate)) AND (((((((Mouth)) OR (Oral Cavity)) OR (Mouth Diseases)) OR (Oral disease)) OR (Tongue)) OR (Tongue disease))) OR (((EGCG) OR (Epigallocatechin-3-gallate)) AND (((((Esophagus) OR (Esophageal Neoplasms)) OR (Esophagus Cancer)) OR (Esophageal Diseases)) OR (Esophagitis)))) OR (((EGCG) OR (Epigallocatechin-3-gallate)) AND (((((((Stomach) OR (Gastric Cancer)) OR (stomach neoplasms)) OR (Stomach Ulcer)) OR (Gastric Ulcer)) OR (Stomach Diseases)) OR (Gastric Diseases)))) OR (((EGCG) OR (Epigallocatechin-3-gallate)) AND ((((Duodenum) OR (Duodenal Neoplasms)) OR (Duodenal Diseases)) OR (Duodenal ulcer)))) OR (((EGCG) OR (Epigallocatechin-3-gallate)) AND (((Jejunum) OR (Jejunal Neoplasms)) OR (Jejunal Diseases)))) OR (((EGCG) OR (Epigallocatechin-3-gallate)) AND (((Ileum) OR (Ileal Neoplasms)) OR (Ileal Diseases)))) OR (((EGCG) OR (Epigallocatechin-3-gallate)) AND ((((((((((Colon) OR (Colonic Neoplasms)) OR (Sigmoid Diseases)) OR (Colonic Diseases)) OR (Colonic cancer)) OR (Colitis)) OR (Colitis-Associated Neoplasms)) OR (Crohn Disease)) OR (Colitis, Ulcerative)) OR (Irritable Bowel Syndrome)))) OR (((EGCG) OR (Epigallocatechin-3-gallate)) AND (((Rectum) OR (Rectal Neoplasms)) OR (Rectal disease)))) OR (((EGCG) OR (Epigallocatechin-3-gallate)) AND (((((((((Liver) OR (Liver disease)) OR (Non-alcoholic Fatty Liver Disease)) OR (Liver Neoplasms)) OR (Hepatic Cancer)) OR (Hepatocellular Cancer)) OR (Fatty Liver)) OR (Hepatitis)) OR (Hepatic Fibrosis)))) OR (((EGCG) OR (Epigallocatechin-3-gallate)) AND ((((((Pancreas) OR (Pancreatic Neoplasms)) OR (Pancreas Cancer)) OR (Pancreatic Cancer)) OR (Pancreatitis)) OR (Pancreatic Diseases)))) OR ((((Toxicology) OR (Toxicity)) OR (Side effects)) AND ((EGCG) OR (Epigallocatechin-3-gallate)))) OR (((((((((Salivary Glands) OR (Gland, Salivary)) OR (Submandibular Gland)) OR (Gland, Submandibular)) OR (Parotid Gland)) OR (Gland, Parotid)) OR (Sublingual Gland)) OR (Gland, Sublingual)) AND ((EGCG) OR (Epigallocatechin-3-gallate)))) OR (((((Gastrointestinal Tract) OR (Digestive Tract)) OR (Digestive System)) OR (digestive glands)) AND ((EGCG) OR (Epigallocatechin-3-gallate)))

**Web of science**

((((((((((((((EGCG) OR (Epigallocatechin-3-gallate)) AND (((((((Mouth)) OR (Oral Cavity)) OR (Mouth Diseases)) OR (Oral disease)) OR (Tongue)) OR (Tongue disease))) OR (((EGCG) OR (Epigallocatechin-3-gallate)) AND (((((Esophagus) OR (Esophageal Neoplasms)) OR (Esophagus Cancer)) OR (Esophageal Diseases)) OR (Esophagitis)))) OR (((EGCG) OR (Epigallocatechin-3-gallate)) AND (((((((Stomach) OR (Gastric Cancer)) OR (stomach neoplasms)) OR (Stomach Ulcer)) OR (Gastric Ulcer)) OR (Stomach Diseases)) OR (Gastric Diseases)))) OR (((EGCG) OR (Epigallocatechin-3-gallate)) AND ((((Duodenum) OR (Duodenal Neoplasms)) OR (Duodenal Diseases)) OR (Duodenal ulcer)))) OR (((EGCG) OR (Epigallocatechin-3-gallate)) AND (((Jejunum) OR (Jejunal Neoplasms)) OR (Jejunal Diseases)))) OR (((EGCG) OR (Epigallocatechin-3-gallate)) AND (((Ileum) OR (Ileal Neoplasms)) OR (Ileal Diseases)))) OR (((EGCG) OR (Epigallocatechin-3-gallate)) AND ((((((((((Colon) OR (Colonic Neoplasms)) OR (Sigmoid Diseases)) OR (Colonic Diseases)) OR (Colonic cancer)) OR (Colitis)) OR (Colitis-Associated Neoplasms)) OR (Crohn Disease)) OR (Colitis, Ulcerative)) OR (Irritable Bowel Syndrome)))) OR (((EGCG) OR (Epigallocatechin-3-gallate)) AND (((Rectum) OR (Rectal Neoplasms)) OR (Rectal disease)))) OR (((EGCG) OR (Epigallocatechin-3-gallate)) AND (((((((((Liver) OR (Liver disease)) OR (Non-alcoholic Fatty Liver Disease)) OR (Liver Neoplasms)) OR (Hepatic Cancer)) OR (Hepatocellular Cancer)) OR (Fatty Liver)) OR (Hepatitis)) OR (Hepatic Fibrosis)))) OR (((EGCG) OR (Epigallocatechin-3-gallate)) AND ((((((Pancreas) OR (Pancreatic Neoplasms)) OR (Pancreas Cancer)) OR (Pancreatic Cancer)) OR (Pancreatitis)) OR (Pancreatic Diseases)))) OR ((((Toxicology) OR (Toxicity)) OR (Side effects)) AND ((EGCG) OR (Epigallocatechin-3-gallate)))) OR (((((((((Salivary Glands) OR (Gland, Salivary)) OR (Submandibular Gland)) OR (Gland, Submandibular)) OR (Parotid Gland)) OR (Gland, Parotid)) OR (Sublingual Gland)) OR (Gland, Sublingual)) AND ((EGCG) OR (Epigallocatechin-3-gallate)))) OR (((((Gastrointestinal Tract) OR (Digestive Tract)) OR (Digestive System)) OR (digestive glands)) AND ((EGCG) OR (Epigallocatechin-3-gallate)))**Supplementary fig. 1.** The chemical information of EGCG.


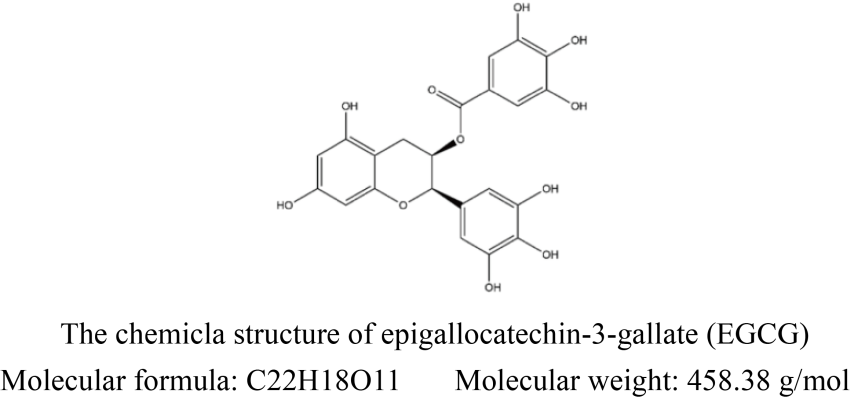


**Supplementary fig. 2.** Forest plot showing the association between EGCG and tumor weight in oral cancer xenograft models.


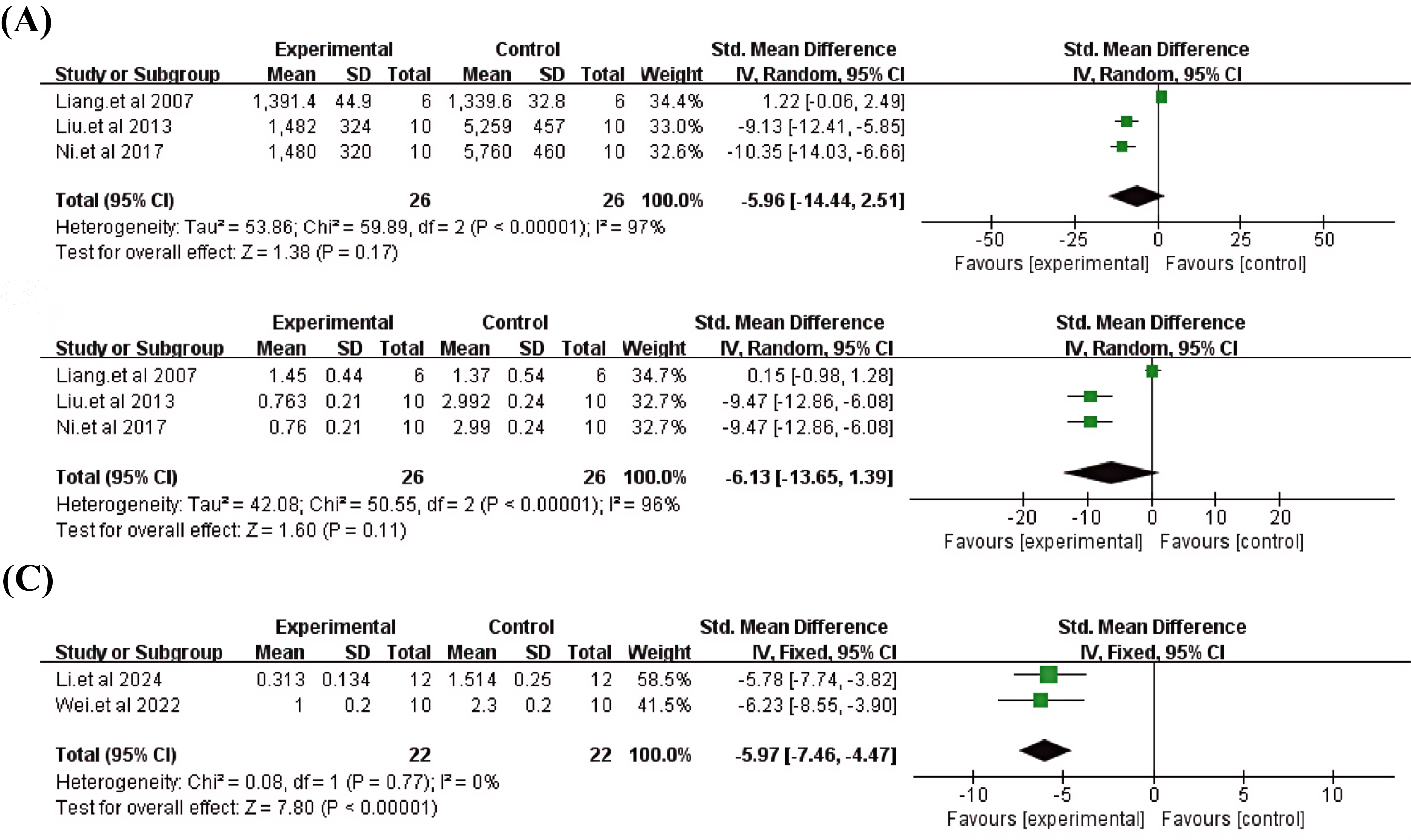


Tumor weight in oral cancer xenograft models (fixed-effects model): SMD = -6.13 (95% CI: -13.65, 1.39);

Effect sizes (SMD) and 95% CIs for each study are shown as squares (size proportional to study weight) and horizontal lines. The pooled effect size is represented by a diamond. KB refers to a human oral epidermoid carcinoma cell line.

**Supplementary fig. 3.** Forest plot showing the association between EGCG and tumor size in gastric cancer models.


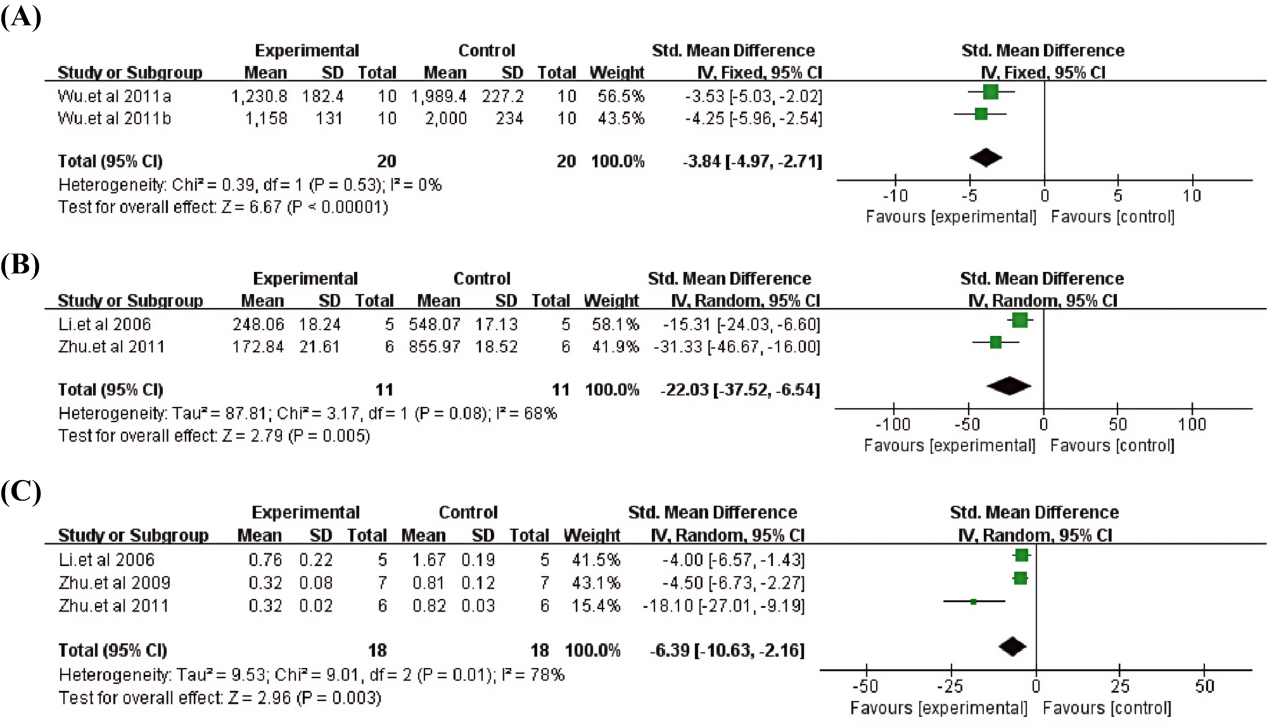


(A) Tumor volume in BGC-823 xenograft models (random‑effects model): SMD = -3.84 (95% CI: -4.97, -2.71);

(B) Tumor volume in SGC7901 xenograft models (random‑effects model): SMD = -22.03 (95% CI: -37.52, -6.54);

(C) Tumor weight in SGC7901 xenograft models (random‑effects model): SMD = -6.39 (95% CI: -10.63, -2.16).

Effect sizes (SMD) and 95% CIs for each study are shown as squares (size proportional to study weight) and horizontal lines. The pooled effect size is represented by a diamond. BGC-823 and SGC7901 are human gastric cancer cell lines.

**Supplementary fig. 4**. Forest plot showing the association between EGCG and tumor size in colorectal cancer models.


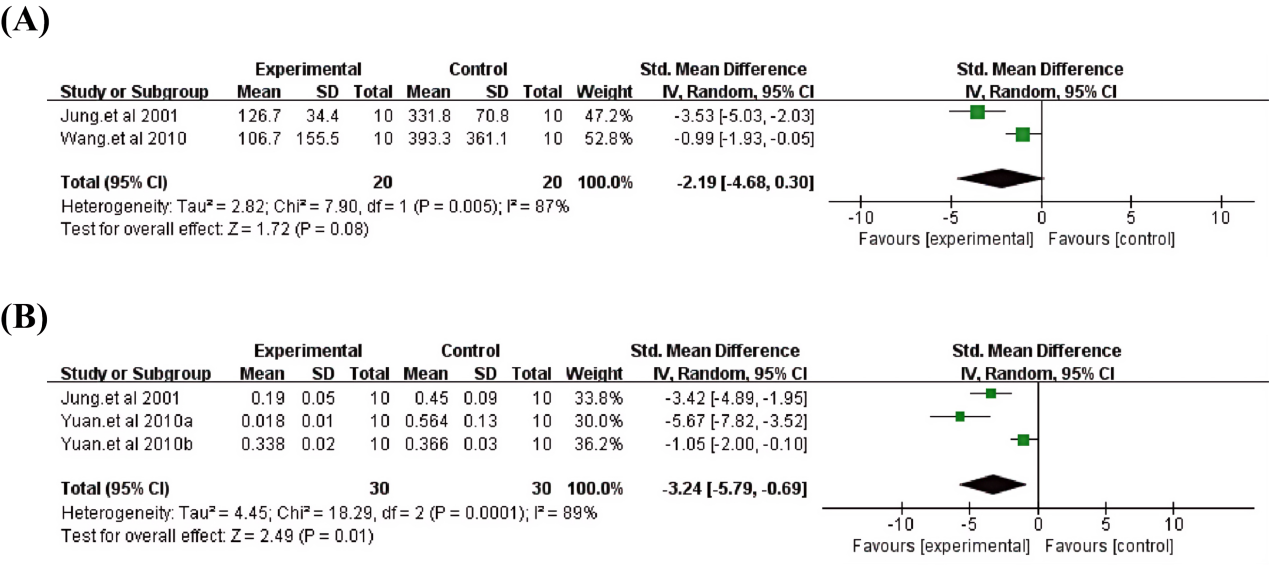


(A) Tumor volume in HT29 xenograft models (random‑effects model): SMD = -2.19 (95% CI: -4.68, 0.30);

(B) Tumor weight in HT29 xenograft models (random‑effects model): SMD = -0.65 (95% CI: -3.24, -0.69).

Effect sizes (SMD) and 95% CIs for each study are shown as squares (size proportional to study weight) and horizontal lines. The pooled effect size is represented by a diamond. HT29 is a human colorectal adenocarcinoma cell line.

**Supplementary fig. 5.** Forest plot showing the association between EGCG and tumor size in gastric cancer models.


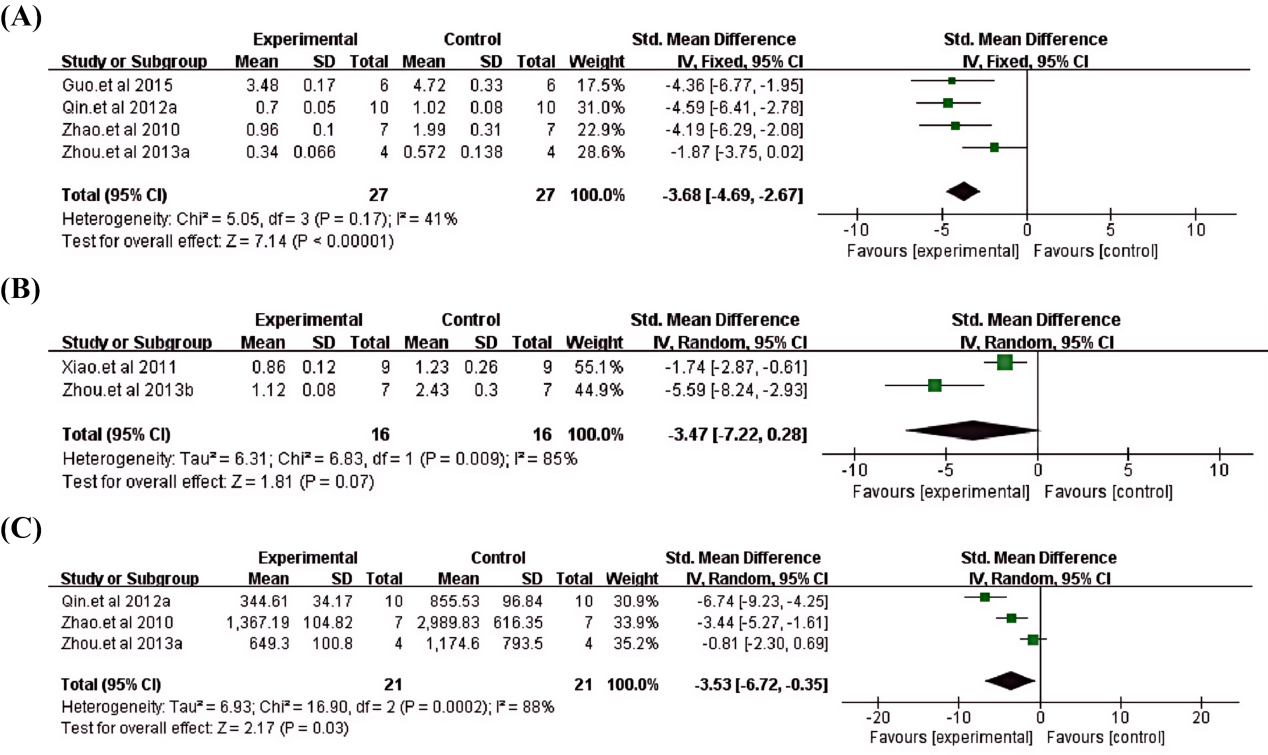


(A) Tumor weight in HepG2 xenograft models (fixed‑effects model): SMD = -3.68 (95% CI: -4.69, -2.67]);

(B) Tumor weight in SMMC-7721 xenograft models (random‑effects model): SMD = -3.47 (95% CI: -7.22, 0.28);

(C) Tumor volume in HepG2 xenograft models (random‑effects model): SMD = -3.53 (95% CI: -6.72, -0.35]).

Effect sizes (SMD) and 95% CIs for each study are shown as squares and horizontal lines; the diamond represents the pooled effect size. A random-effects model was used. Note: HepG2 and SMMC-7721 are human hepatocellular carcinoma cell lines, which have been used as experimental models for gastric cancer in the included studies (as referenced). HT29 is a human colorectal adenocarcinoma cell line.

**Supplementary fig. 6.** Forest plot showing the association between EGCG and MVD in xenograft models.


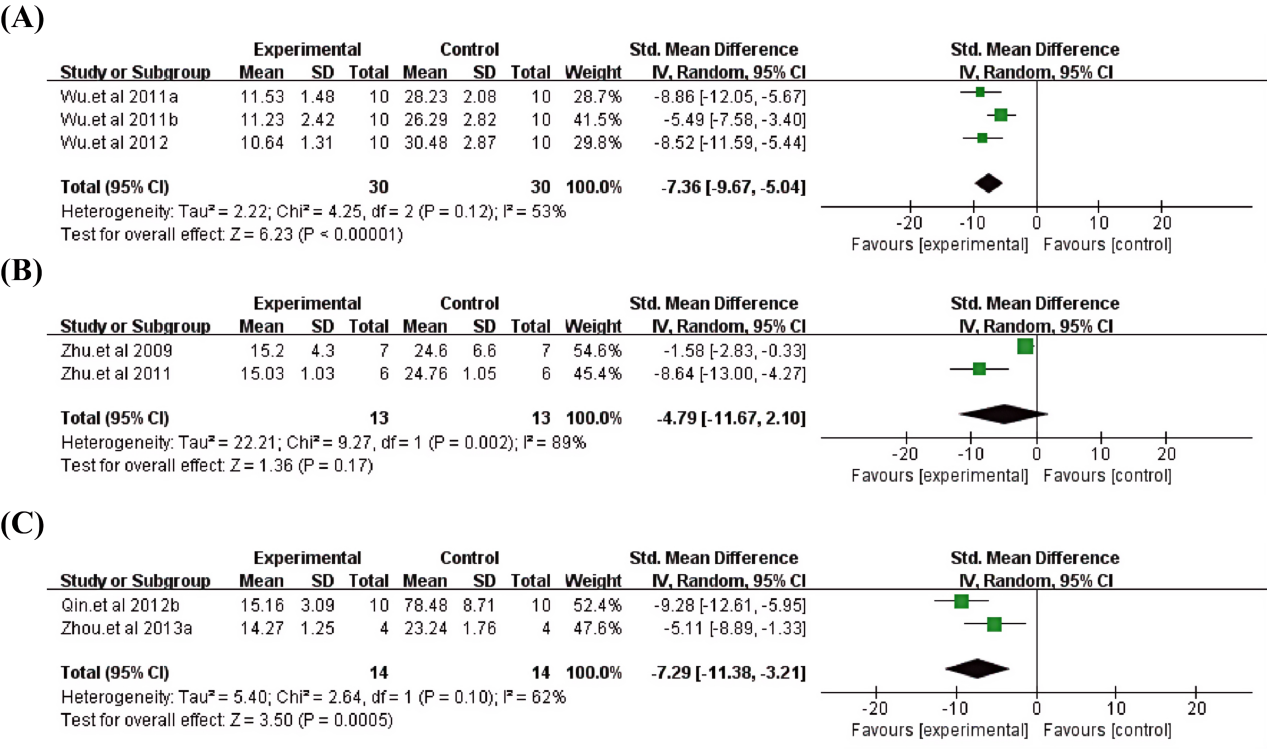


(A) BGC-823 xenograft models (random‑effects model): SMD = -7.36 (95% CI: -9.67, -5.04);

(B) SGC7901 xenograft models (random‑effects model): SMD = -4.79 (95% CI: -11.67, 2.10);

(C) HepG2 xenograft models (random‑effects model): SMD = -7.29 (95% CI: -11.38, -3.21).

Effect sizes (SMD) and 95% CIs for each study are shown as squares and horizontal lines; the diamond represents the pooled effect size. Heterogeneity is indicated by I² statistics shown in each panel. BGC-823 and SGC7901 are human gastric cancer cell lines, and HepG2 is a human hepatocellular carcinoma cell line.
